# Virtual Surgical Planning and Three-Dimensional Models for Precision Sinonasal and Skull Base Surgery

**DOI:** 10.3390/cancers15204989

**Published:** 2023-10-14

**Authors:** Conall W. Fitzgerald, Mohammad Hararah, Tim Mclean, Robbie Woods, Snjezana Dogan, Viviane Tabar, Ian Ganly, Evan Matros, Marc A. Cohen

**Affiliations:** 1 Department of Surgery, Head & Neck Division, Memorial Sloan Kettering Cancer Center, 1275 York Avenue, New York, NY 10065, USA; cofitzgerald@stjames.ie (C.W.F.);; 2 Department of Plastic & Microvascular Reconstructive Surgery, Memorial Sloan Kettering Cancer Center, 1275 York Avenue, New York, NY 10065, USA; 3 Department of Pathology, Memorial Sloan Kettering Cancer Center, 1275 York Avenue, New York, NY 10065, USA; dogans@mskcc.org; 4 Department of Neurosurgery, Memorial Sloan Kettering Cancer Center, 1275 York Avenue, New York, NY 10065, USA

**Keywords:** head and neck surgery, skull base surgery, Microvascular Reconstruction

## Abstract

**Simple Summary:**

Sinonasal and skull base malignancies are rare tumors which present adjacent to numerous critical anatomical structures. Despite the increased use of endoscopic techniques, many patients still require open surgery. Treatment with surgery requires careful pre-operative planning. Virtual surgical planning and three-dimensional, patient-specific models offer a range of benefits to clinicians in terms of planning tumor resection and reconstruction. In additional, these technologies improve communication with patients, trainee doctors and other colleagues. In this article, we explore the applications of virtual surgical planning in sinonasal and skull base surgery.

**Abstract:**

Sinonasal and skull base malignancies represent a rare, heterogenous group of pathologies with an incidence of 0.556 per 100,000 persons in the population. Given the numerous critical anatomic structures located adjacent to the sinonasal cavity and skull base, surgery for tumors in this region requires careful pre-operative planning with the assistance of radiological imaging and intraoperative image guidance technologies to reduce the risk of complications. Virtual surgical planning (VSP) and three-dimensional models (3DMs) are adjunctive technologies which assist clinicians to better visualize patient anatomy using enhanced digital radiological images and physical stereolithographic models based on patients’ personal imaging. This review summarizes our institutional experience with VSP and 3DMs in sinonasal and skull base surgical oncology. A clinical case series is used to thematically illustrate the application of VSP and 3DMs in surgical ablation, reconstruction, patient communication, medical education, and interdisciplinary teamwork in sinonasal and skull base surgery.

## 1. Introduction

Sinonasal and skull base malignancies represent a rare, heterogenous group of pathologies with an incidence of 0.556 per 100,000 persons in the population [1]. Given the numerous critical anatomic structures located adjacent to the sinonasal cavity and skull base, surgery for tumors in this region requires careful pre-operative planning with the assistance of radiological imaging and intraoperative image guidance technologies to reduce the risk of complications [2]. Despite the introduction of minimally invasive, endoscopic surgical techniques, open craniofacial or cranioendoscopic surgery remains an integral technique in managing sinonasal and skull base malignancies [3]. Additional novel technologies now exist to guide surgeons in performing complex sinonasal and skull base procedures.

Virtual surgical planning (VSP) and three-dimensional models (3DMs) are adjunctive technologies which assist clinicians to better visualize patient anatomy using enhanced digital radiological images and physical stereolithographic models based on patients’ personal imaging [4]. Three-dimensional printing of medical imaging was first developed in the late 1970s and early 1980s, and has seen a rapid evolution in recent years with numerous novel clinical, educational, and research applications [5]. By applying computer-aided design and computer-aided manufacturing (CAD-CAM) technology to actual patient imaging, high-fidelity operative models and precision cutting guides can be designed to plan bony incisions and resection margins intraoperatively.

While the use of VSP and 3DMs has been explored in detail in maxillofacial, orthopedic, and craniofacial surgery, fewer reports have described its application in open or endoscopic sinonasal and skull base surgery. In addition, discussion of its role in guiding ablation in sinonasal and skull base surgery and patient communication for these diseases is more limited. This review summarizes our institutional experience with VSP and 3DMs in sinonasal and skull base surgical oncology. A clinical case series is used to thematically illustrate the application of VSP and 3DMs in surgical ablation, reconstruction, patient communication, medical education, and interdisciplinary teamwork in sinonasal and skull base surgery.

## 2. Materials and Methods

A retrospective review of institutional medical records between 2019 and 2021 was performed to identify cases where 3DMs and VSP were utilized during surgery for sinonasal or skull base malignancies with or without microvascular free flap reconstruction (*n* = 280). Cases were excluded where radiological imaging, VSP digital plans, or adequate clinical information were not available. Institutional Review Board approval was granted under an existing protocol to complete this study and written consent was obtained from all patient participants. Patients were staged using the 8th edition of the AJCC [6].

Our institutional practice is to use 3DMs and VSP based on clinical need where the technology is expected to aid interdisciplinary surgical planning, reconstruction, communication, or education. Patients planned for sinonasal and skull base surgery undergo a thorough diagnostic assessment, including clinical examination and multiplanar radiologic assessment with fine-cut computed tomography (CT) and magnetic resonance imaging (MRI), with or without positron emission tomography (PET). All cases are discussed at a skull base multidisciplinary tumor board meeting prior to the initiation of treatment.

The VSP imaging and 3DMs used for surgical planning are created using multiplanar (axial, coronal, and sagittal) CT imaging using 0.5–1.25 mm slice thickness, which is converted into de-identified Digital Imaging and Communications in Medicine (DICOM) format with a 0.33 mm × 0.45 mm pixel size. Manufacture of the 3DM is typically completed by DePuy Synthes (Synthes^®^ Maxillofacial, West Chester, PA, USA) or an alternative agent. A formal meeting between the operating surgeons (all experienced skull base surgeons) and a company representative is completed to review the VSP images and design surgical models and cutting guides pre-operatively. In select cases, alternative, reserve cutting guides are designed to permit multiple ablative and reconstructive options based on the intraoperative clinical findings. Reconstructive materials can be digitally superimposed on the model pre-operatively in order to plan precise reconstruction of the surgical defect. A final report detailing the planned cutting guides is presented to the team pre-operatively. In cases where cutting guides for osteotomy are not required, a 3DM may be produced to guide patient communication, education, or planning.

## 3. Results

### 3.1. Thematic Case Series

#### 3.1.1. Case 1: Application of a 3D Model in Sinonasal and Skull Base Tumor Excision/Ablation

**Figure 1:**
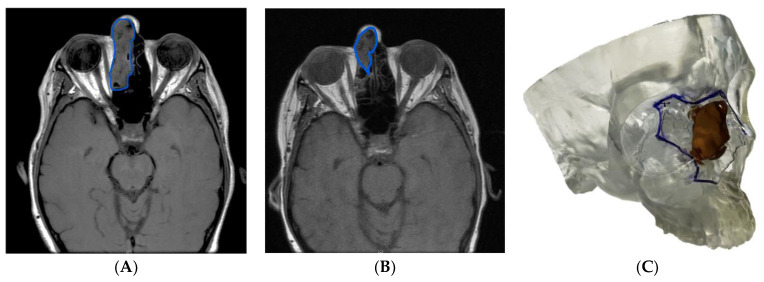
(**A**) T1-weighted axial MRI pre-induction chemotherapy (blue outline highlighting tumor), (**B**) T1-weighted axial MRI post-induction chemotherapy, and (**C**) 3DM showing residual tumor and bony anatomy with planned resection margins.

In illustrative **Case 1**, a 61-year-old female with a cT4aN0M0 ethmoid sinus squamous cell carcinoma (SCC) involving the lacrimal duct received induction chemotherapy prior to open craniofacial resection incorporating nasal bones and medial maxilla. Figure 1A,B demonstrate T1-weighted MRI imaging pre- and post-induction chemotherapy. Induction chemotherapy was delivered to spare critical structures (such as the orbit and brain) but also to maximize cosmetic and functional outcomes by reducing the extent of midface bone and skin resection required. Figure 1C shows the 3DM post-induction chemotherapy. The planned osteotomies were marked to outline the limits of the required resection. This model was then repeatedly referenced intraoperatively to ensure that the ablation followed the pre-surgical plan precisely and adequate oncologic margins could be guaranteed. Complete, en bloc resection with negative margins was achieved in Case 1 with no post-operative complications reported.

Traditionally, to ensure safe and comprehensive tumor excision, anatomical landmarks based on radiological imaging are used to guide ablation intraoperatively. The surgeon will use two-dimensional (2D) images to understand the anatomic relationships between a tumor and its local anatomy and derive a mental 3D plan. This is combined with the use of intraoperative image guidance to maximize surgical accuracy. The use of VSP and a 3DM permits precise planning and demonstration of incision margins on a physical model based on patient imaging in advance of surgery, which may be particularly useful where anatomy is distorted or obliterated by the tumor. Partial response to induction chemotherapy, pre-operative radiation therapy, and a non-centripetal pattern of tumor response may make recognizing anatomical landmarks more challenging, further strengthening the case for the use of VSP as an aid during surgery. The authors highlight the use of 3DMs to provide precise cutting guides for bony cuts in the midface, which may serve to enhance the quality and accuracy of reconstruction. 3D-printed cutting guides may or may not be used based on tumor location and the ability to affix cutting guides to bone.

#### 3.1.2. Case 2: Employing a 3D Model to Enhance Patient Communication

**Case 2** describes an 8-year-old male with a diagnosis of a cT2N0M0 nasopharyngeal embryonal rhabdomyosarcoma treated with chemotherapy and radiotherapy. The patient subsequently developed local recurrence high in the parapharyngeal space closely applied to both the internal carotid artery and external carotid artery branches. In this case, a 3DM and VSP allowed a greater appreciation of the relationship of the tumor and major arteries when communicating the need for preoperative embolization of the proximal internal carotid artery, the internal carotid at the foramen lacerum, and the maxillary branch of the external carotid artery to the patient’s family. This permitted a lip-split approach to en bloc removal of the tumor encompassing the ramus of the mandible, the masticator space, and the parapharyngeal space.

**Figure 2:**
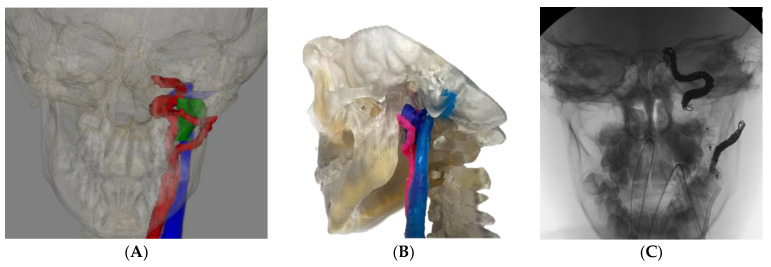
(**A**) 3D radiologic rendering of a tumor and related bony and vascular structures highlighted the carotid artery (red), internal jugular vein (blue) and tumor (green), (**B**) 3D model of the tumor with highlighted vascular structures, and (**C**) image of pre-operative embolization of the left carotid system.

Given the rarity and anatomical complexity of tumors in the sinonasal and skull base region, educating patients and their families regarding treatment plans, alternative options, and the associated risks is essential. Frequently, however, this presents a conceptual challenge for patients, who may be presented with 2D radiological imaging during discussion. In this type of case, a 3D rendered image (Figure 2A) can be used as a communication tool to highlight the complexities of this case to the patient and their family. A 3DM (Figure 2B) was created to guide surgical planning and assist in decision-making and informed consent. This model highlighted the proximity of the internal jugular vein and carotid artery to explain the requirement for embolization, which is shown in Figure 2C.

The use of a 3DM in this case assisted understanding of the need for a complex multi-stage treatment process. Improving communication and patient education is one way in which physicians can hope to improve patient satisfaction and outcomes using VSP and 3DMs.

#### 3.1.3. Case 3: Use of Virtual Surgical Planning and a 3D Model in Resident and Fellow Education

**Figure 3:**
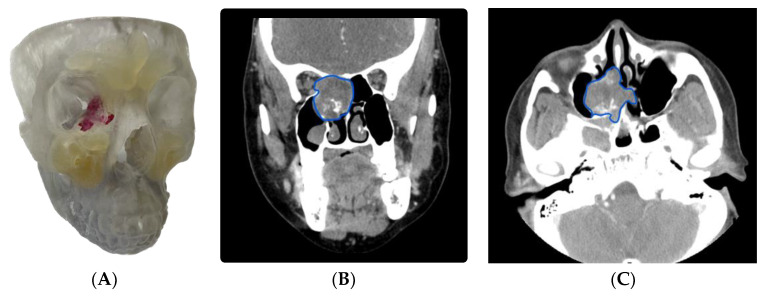
(**A**) 3DM rendering of the tumor and related bony and vascular structures, (**B**) coronal CT highlighting the complexity of the tumor location (blue outline highlighting tumor), and (**C**) axial CT image highlighting the tumor’s relationship to the petroclival internal carotid artery.

**Case 3** describes a 69-year-old female who presented with nasal obstruction, facial pain, swelling, and nasal cavity mass. A complete workup confirmed a cT4aN0M0 ethmoid sinus adenoid cystic carcinoma with invasion of the maxillary sinus, sphenoid sinus, and anterior cranial fossa, with abutment of the internal carotid artery (Figure 3B,C). The patient was enrolled in a combined-modality prospective trial and treated with transnasal endoscopic surgical resection with nasoseptal flap and layered fascial graft reconstruction, followed by adjuvant chemoradiotherapy.

Surgical residents and fellows involved in the workup, surgical management, and aftercare of patients with sinonasal and skull base tumors can benefit from the use of VSP and 3DMs (Figure 3A). Trainees are required to understand the anatomical landmarks needed to guide safe surgery. The use of a 3D, life-size replica of the patient’s anatomy, in combination with radiological images, may advance trainee progression along the learning curve, particularly in surgeries where training opportunities may be more limited due to case complexity or rarity. In addition, these technologies may reduce the requirement for cadaveric specimens in training.

Using the 3DM, residents and fellows were able to directly visualize the tumor extent and potential pitfalls of the endoscopic technique employed. This permitted them to assist in complete resection of the tumor with negative margins. Sound anatomical knowledge and competent surgical skills remain the most critical skills for trainees, with VSP and 3DM technology offering great potential to enhance resident and fellow education.

#### 3.1.4. Case 4: The Role of Virtual Surgical Planning and a 3D Model in Complex Sinonasal Reconstruction

**Figure 4:**
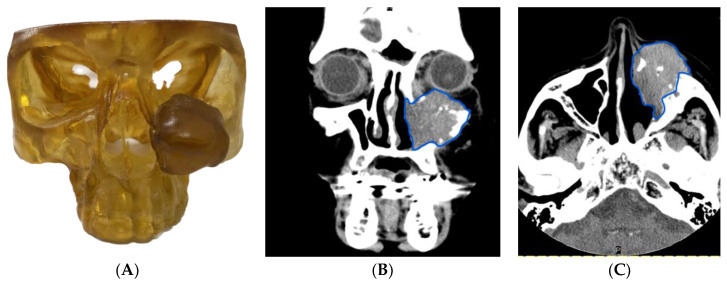
(**A**) 3DM of the patient’s tumor demonstrating the extent of local invasion, (**B**) coronal CT image demonstrating the extent of inferior tumor invasion (blue outline highlighting tumor), and (**C**) axial MRI image highlighting the posterior and medial extent of tumor invasion.

In **Case 4**, an 87-year-old female presented with a new diagnosis of a cT4aN0M0 left maxillary sinus SCC. The patient previously underwent multiple surgical resections for an inverted sinus papilloma at outside institutions and presented to our center following rapid expansion of the mass. The imaging highlighted an expansile, multicompartment maxillary sinus tumor measuring 5.5 cm × 3.0 cm, involving the infraorbital nerve and the floor of the orbit, with extraconal orbital spread and infiltration of all the walls of the maxillary sinus (Figure 4B,C).

The patient underwent total maxillectomy with reconstruction with a vertical rectus abdominis myocutaneous free flap and a titanium mesh to reconstruct the orbital floor. VSP and a 3DM were used in this case to demonstrate the anticipated osteotomies and the resulting defect to the reconstructive team in advance of surgery (Figure 4A). Using the described workflow, fine-cut CT images were reviewed at a surgical planning meeting where the anticipated defect could be measured and discussed. Reconstruction could then be planned, including for potential use of bone grafting, pre-operative shaping of titanium plates based on the 3DM, and sizing of the myocutaneous free flap. Given the large size of the anticipated defect, use of a flap offering a large myogenous component (in this case a rectus abdominis flap) was opted for, which also offered the additional benefit of a cutaneous lining for the nasal cavity.

Use of VSP in this case was focused on assessing the best possible reconstructive options for this elderly patient and reducing the operative time while enhancing the patient’s functional and cosmetic outcomes. Further potential advantages of VSP and 3DMs are the ability to plan prosthodontic rehabilitation pre-operatively as well as applying cutting guides not only for the ablative osteotomies but also the reconstructive osteotomies, for example, in scapular or fibular free flap reconstruction. This patient underwent successful resection and reconstruction without complication.

#### 3.1.5. Case 5: The Role of Virtual Surgical Planning and a 3D Model in Multidisciplinary Teamwork

**Figure 5:**
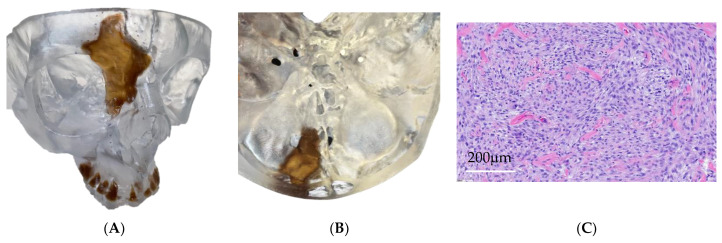
(**A**,**B**) Anterior and superior views of patient’s 3DM demonstrating the extent of anterior skull base invasion and (**C**) post-operative histopathology H&E stain demonstrating the pattern of spindle cell/sclerosing rhabdomyosarcoma.

**Case 5** describes a 54-year-old female presenting with a cT4aN0M0 spindle cell sclerosing rhabdomyosarcoma involving the anterior skull base, ethmoid sinus, frontal sinus, and orbit (Figure 5A,B). Treatment with induction chemotherapy and proton beam radiation failed to reduce the extent of the tumor and open craniofacial resection was planned. A 3DM was made and VSP undertaken to facilitate operative planning, which involved multiple teams, including neurosurgery, head and neck surgery, oculoplastic surgery, and plastic and reconstructive surgery. A collaborative surgical planning meeting was held to plan the optimal operative approach to achieve safe complete resection with negative margins. The VSP and 3DM formed an integral part of the discussion, permitting hands-on assessment of the tumor and measurement of the required margins and anticipated defect. The critical adjacent vascular structures were mapped and decisions to preserve structures, such as the floor of the orbit and contralateral frontal sinus, could be made.

An open craniofacial resection with skull base reconstruction using a peri-cranial flap, calvarial bone graft, and radial forearm free tissue transfer was ultimately undertaken (Figure 5C). The 3DM was consulted intraoperatively to plan the surgical steps and ensure the operative plan was precisely followed. The defect and the extirpated specimen were compared to the 3DM to ensure the completeness of ablation and the accuracy of the intended reconstructive plan. Finally, post-operatively, the tumor specimen and 3DM were then reviewed together with the pathologist to assist with orientation and pathologic assessment. The use of 3DMs and VSP can form an integral part of operative planning and interdisciplinary communication in complex multi-stage open craniofacial resection.

## 4. Discussion

We describe the role of VSP and 3DMs in sinonasal and skull base surgery with a thematic clinical case series demonstrating real-world applications in surgical ablation, reconstruction, resident and fellow education, and multidisciplinary teamwork. We particularly highlight the role of 3DMs and VSP in providing potentially more precise oncologic resection and more comprehensive planning for reconstruction. Additionally, the role of VSP and 3DMs in enhancing patient understanding of surgery and improving communication between team members has been discussed as additional benefits of these technologies in the setting of sinonasal and skull base surgery.

The role of VSP and 3DMs in mapping tumor extent pre-operatively and to ensure adequate oncologic resection has been established in head and neck surgery, as well as other specialties, but is less completely explored in the skull base field [7,8,9]. As demonstrated in our series, the use of 3DMs can assist surgeons in sparing normal structures, particularly in areas altered by malignancy or previous treatment. In the authors experience, the use of VSP and 3DMs can improve margin control and increase the likelihood of achieving en bloc resection with negative margins, particularly when “blind” cuts are required, where the tumor cannot be visualized externally. In specific anatomic regions without reliable surface landmarks to guide bony incisions, such as the midface or frontal sinus, this technology offers particular benefits. Additional advantages of VSP and 3DMs described in the literature include a reduced task workload (the effort required to complete surgery), improvements in surgical workflows, reductions in exposure to ionizing radiation (where used intraoperatively), and improved post-operative patient outcomes [10,11].

Precision cutting guides modelled on VSP have been validated in maxillofacial and craniofacial surgery [12]. It is the authors’ opinion that the relative rarity of sinonasal and skull base tumors may be the primary cause for slow adoption of cutting guides in sinonasal surgery. Other challenges include affixing cutting guides to the anatomy targeted during skull base surgery. The authors propose that cutting guides created using VSP and 3DM technologies may currently be underutilized in the field of sinonasal and skull base surgery and may offer benefits in a range of cases. The technology may also assist with mapping critical vascular structures and enhancing negative margin rates; a reduced surgical time, reduced overall complication rates, and a reduced workload have also been reported [13,14,15].

We highlight the role of 3DMs and VSP in bony and soft tissue microvascular free flap reconstruction of the skull base. Reconstruction of the skull base typically requires multiple forms of tissue to establish adequate functional reconstruction without CSF leak or other complications. We demonstrate in our series that estimating the size of the ablative defect more precisely using VSP in advance of surgery permits improved reconstruction, as seen in Case 4. We highlight improved bony contact allowing enhanced anatomical reconstruction where VSP/a 3DM is used to plan osteotomies, as well as the possibility to assess nasal anatomy and vasculature for nasoseptal flap reconstruction [16,17]. Outside the skull base, the role of VSP and 3DMs with cutting guides in planning bony reconstruction is well established, with increased reconstructive accuracy, reduced operative time, and reduced ischemic time described [18,19,20,21,22]. By providing more accurate pre-operative images of the tumor and anticipated defect, reconstructive teams can better plan the reconstruction required, pre-fabricate reconstructive plates, and manufacture cutting guides. Additionally, the information provided to the patient pre-operatively and description of expected outcomes can be improved with the use of VSP and 3DMs as visual aids [23].

The use of VSP and 3DMs in place of cadaveric specimens has been described in both medical student and resident education [24]. Obtaining and storing cadaveric specimens for educational purposes is costly and presents numerous challenges to training bodies, with 3DMs offering a potential alternative. Sinonasal and temporal bone 3DMs have been described as a training aid for otolaryngology residents and fellows [25,26]. Utilizing 3DMs, together with other technologies such as digital image libraries, can provide an excellent, on-demand educational resource in lieu of gross specimens when teaching difficult anatomical concepts [27].

New technologies have also developed in tandem with VSP and 3DMs. Patient-specific instruments designed to conform to a patient’s particular pathology have been described. Manufacture of 3D-printed bone scaffolds which permit bony ingrowth has been reported [28]. The addition of augmented reality (AR) and virtual reality (VR) technologies permits clinicians to overlay digital images onto intraoperative endoscopic views or to create simulated digital models of the patients’ radiological imaging to assist in surgical planning in complex cases [29]. Dixon et al. have described increased accuracy in identifying critical anatomical landmarks where augmented image guidance is employed, with resulting improved overall patient safety [30].

As with all new technologies, potential disadvantages exist with VSP and 3DMs. Though some authors have demonstrated reduced costs, the cost of VSP and 3DM technology can be significant [31]. Additionally, costs may vary based on suppliers, the location of the hospital, and the required materials for the clinical case, while 3DMs and VSP are not available in all jurisdictions. Efforts are being made to create open-source software to permit VSP in low-income settings [32]. No dedicated guidelines exist regarding when or how VSP and 3DMs should be applied in sinonasal and skull base surgery. The additional time to complete the workflow required for 3DMs and VSP adds to the clinician workload and requires a dedicated meeting of multiple senior decision-makers in the multidisciplinary team [33]. Tumor progression during the time required to create a 3DM is possible; however, production times are now significantly shorter. When applied selectively based on clinical need, the benefits for the patient and operative team are evident. Finally, as discussed, use of a cutting guide may not be physically possible in the constrained skull base space although VSP technologies can still be applied.

## 5. Conclusions

Virtual surgical planning and 3D modeling offer a variety of benefits in sinonasal and skull base surgery to enhance both the quality of surgical ablation and guide reconstruction in these complex surgeries. In addition to their immediate clinical applications, VSP and 3DMs have potential for use as communication and education tools for both patients and trainees. We highlight 3DMs and VSP as useful tools in the future of the multidisciplinary management of sinonasal and skull base tumors.

## Data Availability

The data presented in this study are available in this article.
